# *N*-(Quinolin-8-yl)ferrocene-1-carbox­amide

**DOI:** 10.1107/S1600536811026341

**Published:** 2011-07-23

**Authors:** Xia Li, Ling-Zhi Du

**Affiliations:** a Department of Chemistry and Chemical Engineering, Henan University of Urban Construction, Pingdingshan, Henan 467044, People’s Republic of China

## Abstract

In the title compound, [Fe(C_5_H_5_)(C_15_H_11_N_2_O)], the cyclo­penta­dienyl rings are essentially eclipsed, and the dihedral angle between the cyclo­penta­dienyl ring planes is 0.632 (10)°. The Fe atom is slightly closer to the substituted cyclo­penta­dienyl ring, with an Fe–centroid distance of 1.6374 (3) Å [1.6494 (3) Å for the unsubstituted ring]. The amide group is essentially coplanar with the substituted cyclo­penta­dienyl ring, with an N—C(O)—C—C torsion angle of 2.3 (3)°.

## Related literature

For background to the chemical, stereochemical and electrochemical properties of ferrocene, see: Togni & Hayashi (1995 ▶). Ferrocene has been extensively incorporated into larger compounds in order to take advantage of these properties, see: Abd-El-Aziz & Manners (2007 ▶); Fang *et al.* (2001 ▶); Mata *et al.* (2001 ▶). For our research on ferrocenyl derivatives and their metal complexes, see: Li *et al.* (2008 ▶, 2009 ▶).
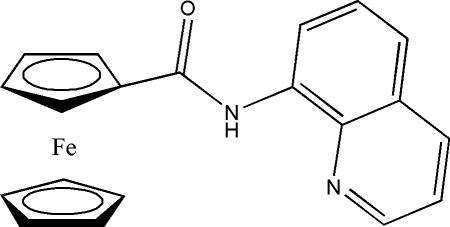


## Experimental

### 

#### Crystal data

[Fe(C_5_H_5_)(C_15_H_11_N_2_O)]
*M* *_r_* = 356.20
Orthorhombic, 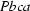

*a* = 10.1680 (17) Å
*b* = 12.133 (2) Å
*c* = 26.079 (4) Å
*V* = 3217.4 (10) Å^3^
*Z* = 8
Mo *K*α radiation
μ = 0.95 mm^−1^
*T* = 296 K
0.46 × 0.37 × 0.25 mm

#### Data collection

Rigaku Mercury CCD diffractometer
Absorption correction: multi-scan (*CrystalClear*; Rigaku, 2000 ▶) *T*_min_ = 0.668, *T*_max_ = 0.795
18658 measured reflections
3940 independent reflections
2996 reflections with *I* > 2σ(*I*)
*R*_int_ = 0.031

#### Refinement

*R*[*F* ^2^ > 2σ(*F* ^2^)] = 0.034
*wR*(*F* ^2^) = 0.097
*S* = 1.01
3940 reflections
222 parameters
H-atom parameters constrained
Δρ_max_ = 0.26 e Å^−3^
Δρ_min_ = −0.37 e Å^−3^

### 

Data collection: *CrystalClear* (Rigaku, 2000 ▶); cell refinement: *CrystalClear*; data reduction: *CrystalClear*; program(s) used to solve structure: *SHELXS97* (Sheldrick, 2008 ▶); program(s) used to refine structure: *SHELXL97* (Sheldrick, 2008 ▶); molecular graphics: *SHELXTL* (Sheldrick, 2008 ▶); software used to prepare material for publication: *SHELXTL*.

## Supplementary Material

Crystal structure: contains datablock(s) I, global. DOI: 10.1107/S1600536811026341/fj2441sup1.cif

Structure factors: contains datablock(s) I. DOI: 10.1107/S1600536811026341/fj2441Isup2.hkl

Additional supplementary materials:  crystallographic information; 3D view; checkCIF report

## Figures and Tables

**Fig. 1.**
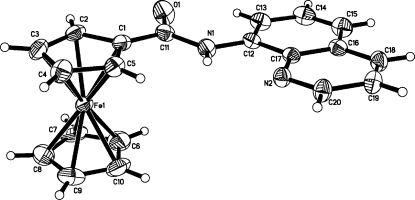
The molecular structure of the title compound, showing the atomic numbering and 30% probability displacement ellipsoids.

## supplementary crystallographic information

### Comment

Due to its special chemical, stereochemical and electrochemical properties (Togni *et al.*, 1995), ferrocene has been extensively incorporated into larger compounds in order to take advantage of these properties (Mata *et al.*, 2001; Abd-El-Aziz *et al.*, 2007; Fang *et al.*, 2001). As a continuation of our research related to ferrocenyl derivatives and their metal complexes (Li *et al.*, 2008; Li *et al.*, 2009), herein we report the crystal structure of 8-(Ferrocenoylamino)quinoline.

The molecular structure of the title compound is composed of a ferrocenyl group and a quinolyl group joined by an organic amide spacer. The Fe—C bond distances within the ferrocene group are in the range of 2.0259 (19)–2.048 (2) Å for the substituted cyclopentadienyl (Cp) ring [C1—C5] and 2.026 (2)–2.039 (2) Å for the unsubstituted Cp ring [C6—C10]. The planar cyclopentadienyl rings of the ferrocenyl unit are nearly parallel to each other [the interplanar angle is 0.632 (10) °]. The Cp rings are essentially eclipsed and the Fe-centroid distances are 1.6374 (3) Å (*Cg*1) and 1.6494 (3) Å (*Cg*2) with *Cg*1 and *Cg*2 are the centroids of the [C1—C5] and [C6—C10] rings. The [*Cg*1—Fe1—*Cg*2] angle is 179.324 (17) °. The carbamoyl group is essentially coplanar with the substituted cyclopentadienyl ring with a deviation of 4.9 (2) °. The angle formed by the carbamoyl group and the quinolyl group system is 9.4 (3) °.

### Experimental

A solution of Chlorocarbonyl ferrocene (0.248 g, 1 mmol) in CH_2_Cl_2_ (20 ml) was added dropwise to a vigorously stirred solution of the 8-Aminoquinoline (0.144 g, 1 mmol) in CH_2_Cl_2_ (20 ml) containing pyridine (0.5 ml). The stirred reaction mixture was maintained at room temperature for 4 h. Removal of the solvent afforded the crude amide and the residue was recrystallized in dichloromethane/ether to give orange crystals 0.325 g. Yield 91.3%.

### Refinement

H atom bonded to N atom was located from difference Fourier maps and refined with a *DFIX* restraint of 0.86 (2) Å. Aromatic H atoms were positioned geometrically with C—H = 0.95 Å and constrained to ride on their parent atoms with *U*_iso_(H) = 1.2*U*_eq_(C).

### Crystal data

**Table:**

[Fe(C_5_H_5_)(C_15_H_11_N_2_O)]	*F*(000) = 1472
*M**_r_* = 356.20	*D*_x_ = 1.471 Mg m^−^^3^
Orthorhombic, *P**b**c**a*	Mo *K*α radiation, λ = 0.71073 Å
Hall symbol: -P 2ac 2ab	θ = 1.6–28.3°
*a* = 10.1680 (17) Å	µ = 0.95 mm^−^^1^
*b* = 12.133 (2) Å	*T* = 296 K
*c* = 26.079 (4) Å	Block, orange
*V* = 3217.4 (10) Å^3^	0.46 × 0.37 × 0.25 mm
*Z* = 8	

### Data collection

**Table:**

Rigaku Mercury CCD diffractometer	3940 independent reflections
Radiation source: fine-focus sealed tube	2996 reflections with *I* > 2σ(*I*)
graphite	*R*_int_ = 0.031
ω scan	θ_max_ = 28.3°, θ_min_ = 1.6°
Absorption correction: multi-scan (*CrystalClear*; Rigaku, 2000)	*h* = −13→13
*T*_min_ = 0.668, *T*_max_ = 0.795	*k* = −12→15
18658 measured reflections	*l* = −34→29

### Refinement

**Table:**

Refinement on *F*^2^	Primary atom site location: structure-invariant direct methods
Least-squares matrix: full	Secondary atom site location: difference Fourier map
*R*[*F*^2^ > 2σ(*F*^2^)] = 0.034	Hydrogen site location: inferred from neighbouring sites
*wR*(*F*^2^) = 0.097	H-atom parameters constrained
*S* = 1.01	*w* = 1/[σ^2^(*F*_o_^2^) + (0.*P*)^2^ + 1.0596*P*] where *P* = (*F*_o_^2^ + 2*F*_c_^2^)/3
3940 reflections	(Δ/σ)_max_ = 0.001
222 parameters	Δρ_max_ = 0.26 e Å^−^^3^
0 restraints	Δρ_min_ = −0.37 e Å^−^^3^

### Special details

**Table:**

Geometry. All e.s.d.'s (except the e.s.d. in the dihedral angle between two l.s. planes) are estimated using the full covariance matrix. The cell e.s.d.'s are taken into account individually in the estimation of e.s.d.'s in distances, angles and torsion angles; correlations between e.s.d.'s in cell parameters are only used when they are defined by crystal symmetry. An approximate (isotropic) treatment of cell e.s.d.'s is used for estimating e.s.d.'s involving l.s. planes.
Refinement. Refinement of *F*^2^ against ALL reflections. The weighted *R*-factor *wR* and goodness of fit *S* are based on *F*^2^, conventional *R*-factors *R* are based on *F*, with *F* set to zero for negative *F*^2^. The threshold expression of *F*^2^ > σ(*F*^2^) is used only for calculating *R*-factors(gt) *etc*. and is not relevant to the choice of reflections for refinement. *R*-factors based on *F*^2^ are statistically about twice as large as those based on *F*, and *R*- factors based on ALL data will be even larger.

### Fractional atomic coordinates and isotropic or equivalent isotropic displacement parameters (Å^2^)

**Table:**

	*x*	*y*	*z*	*U*_iso_*/*U*_eq_	
Fe1	0.08194 (2)	0.61371 (2)	0.324077 (9)	0.03985 (12)	
O1	−0.14938 (18)	0.85691 (16)	0.34733 (7)	0.0757 (5)	
N1	0.02468 (18)	0.87266 (15)	0.40153 (7)	0.0471 (4)	
N2	0.19232 (16)	0.94139 (13)	0.47326 (6)	0.0433 (4)	
C1	−0.04130 (18)	0.69359 (18)	0.37255 (7)	0.0432 (4)	
C2	−0.1135 (2)	0.6191 (2)	0.34083 (8)	0.0509 (5)	
H2	−0.1811	0.6384	0.3186	0.061*	
C3	−0.0656 (2)	0.5119 (2)	0.34881 (8)	0.0580 (6)	
H3	−0.0959	0.4483	0.3328	0.070*	
C4	0.0368 (2)	0.5175 (2)	0.38554 (8)	0.0585 (6)	
H4	0.0856	0.4581	0.3977	0.070*	
C5	0.0521 (2)	0.62850 (18)	0.40057 (7)	0.0484 (5)	
H5	0.1122	0.6549	0.4245	0.058*	
C6	0.1656 (3)	0.7278 (2)	0.27759 (10)	0.0756 (8)	
H6	0.1460	0.8027	0.2772	0.091*	
C7	0.1037 (2)	0.6449 (3)	0.24815 (9)	0.0741 (8)	
H7	0.0355	0.6550	0.2249	0.089*	
C8	0.1644 (3)	0.5444 (3)	0.26050 (10)	0.0736 (8)	
H8	0.1437	0.4758	0.2468	0.088*	
C9	0.2604 (3)	0.5657 (3)	0.29680 (10)	0.0767 (8)	
H9	0.3153	0.5133	0.3117	0.092*	
C10	0.2614 (2)	0.6765 (3)	0.30727 (11)	0.0775 (8)	
H10	0.3171	0.7115	0.3304	0.093*	
C11	−0.06124 (18)	0.81347 (19)	0.37209 (7)	0.0475 (5)	
C12	0.02430 (18)	0.98542 (16)	0.41223 (7)	0.0419 (4)	
C13	−0.0553 (2)	1.0624 (2)	0.38937 (8)	0.0525 (5)	
H13	−0.1144	1.0406	0.3641	0.063*	
C14	−0.0483 (2)	1.1737 (2)	0.40382 (9)	0.0602 (6)	
H14	−0.1021	1.2247	0.3875	0.072*	
C15	0.0351 (2)	1.20882 (19)	0.44113 (9)	0.0583 (5)	
H15	0.0374	1.2829	0.4503	0.070*	
C16	0.1185 (2)	1.13215 (16)	0.46605 (7)	0.0437 (4)	
C17	0.11420 (17)	1.02001 (15)	0.45129 (6)	0.0381 (4)	
C18	0.2047 (2)	1.16091 (17)	0.50584 (8)	0.0506 (5)	
H18	0.2107	1.2338	0.5166	0.061*	
C19	0.2793 (2)	1.08233 (18)	0.52850 (8)	0.0512 (5)	
H19	0.3351	1.1000	0.5555	0.061*	
C20	0.2707 (2)	0.97401 (17)	0.51045 (7)	0.0489 (5)	
H20	0.3241	0.9213	0.5258	0.059*	
H1	0.078 (2)	0.8400 (19)	0.4191 (8)	0.046 (6)*	

### Atomic displacement parameters (Å^2^)

**Table:**

	*U*^11^	*U*^22^	*U*^33^	*U*^12^	*U*^13^	*U*^23^
Fe1	0.03308 (17)	0.0496 (2)	0.03683 (16)	−0.00552 (11)	0.00144 (10)	−0.00378 (11)
O1	0.0693 (11)	0.0826 (12)	0.0752 (11)	0.0229 (10)	−0.0350 (9)	−0.0182 (9)
N1	0.0441 (9)	0.0508 (10)	0.0464 (9)	0.0082 (8)	−0.0110 (7)	−0.0072 (7)
N2	0.0460 (9)	0.0388 (9)	0.0451 (8)	0.0007 (7)	−0.0054 (7)	−0.0007 (7)
C1	0.0344 (9)	0.0619 (13)	0.0334 (8)	−0.0037 (9)	0.0001 (7)	−0.0077 (8)
C2	0.0341 (10)	0.0756 (16)	0.0431 (10)	−0.0137 (10)	0.0019 (8)	−0.0091 (10)
C3	0.0572 (13)	0.0613 (15)	0.0554 (12)	−0.0254 (11)	0.0076 (10)	−0.0071 (11)
C4	0.0674 (14)	0.0551 (13)	0.0530 (12)	−0.0080 (11)	0.0008 (10)	0.0106 (10)
C5	0.0501 (11)	0.0597 (13)	0.0356 (9)	−0.0065 (10)	−0.0038 (8)	0.0002 (9)
C6	0.0774 (18)	0.0681 (17)	0.0813 (17)	−0.0078 (14)	0.0356 (15)	0.0080 (14)
C7	0.0482 (13)	0.133 (3)	0.0411 (11)	−0.0043 (15)	0.0088 (9)	0.0110 (14)
C8	0.0768 (18)	0.0833 (19)	0.0608 (14)	−0.0106 (15)	0.0243 (13)	−0.0214 (13)
C9	0.0494 (14)	0.106 (2)	0.0749 (16)	0.0155 (15)	0.0118 (12)	−0.0030 (16)
C10	0.0445 (13)	0.113 (3)	0.0748 (16)	−0.0260 (15)	0.0118 (12)	−0.0170 (16)
C11	0.0408 (10)	0.0643 (14)	0.0374 (9)	0.0060 (9)	−0.0037 (8)	−0.0098 (9)
C12	0.0406 (10)	0.0467 (11)	0.0385 (9)	0.0054 (8)	0.0056 (7)	−0.0018 (8)
C13	0.0492 (12)	0.0625 (14)	0.0457 (10)	0.0110 (10)	−0.0004 (9)	0.0057 (10)
C14	0.0623 (14)	0.0544 (14)	0.0640 (13)	0.0192 (11)	0.0068 (11)	0.0163 (11)
C15	0.0645 (14)	0.0443 (12)	0.0660 (13)	0.0077 (11)	0.0078 (11)	0.0087 (10)
C16	0.0476 (10)	0.0360 (10)	0.0475 (10)	0.0009 (8)	0.0121 (8)	0.0050 (8)
C17	0.0390 (9)	0.0388 (10)	0.0366 (8)	0.0017 (8)	0.0064 (7)	0.0027 (7)
C18	0.0603 (13)	0.0368 (10)	0.0548 (11)	−0.0077 (9)	0.0087 (10)	−0.0059 (9)
C19	0.0599 (13)	0.0455 (11)	0.0483 (10)	−0.0104 (10)	−0.0063 (9)	−0.0018 (9)
C20	0.0562 (12)	0.0390 (10)	0.0515 (10)	−0.0009 (9)	−0.0102 (9)	0.0011 (8)

### Geometric parameters (Å, °)

**Table:**

Fe1—C10	2.026 (2)	C6—C10	1.391 (4)
Fe1—C5	2.0258 (19)	C6—C7	1.413 (4)
Fe1—C1	2.0268 (19)	C6—H6	0.9300
Fe1—C6	2.028 (2)	C7—C8	1.404 (4)
Fe1—C7	2.028 (2)	C7—H7	0.9300
Fe1—C9	2.034 (2)	C8—C9	1.384 (4)
Fe1—C4	2.036 (2)	C8—H8	0.9300
Fe1—C2	2.036 (2)	C9—C10	1.372 (4)
Fe1—C8	2.039 (2)	C9—H9	0.9300
Fe1—C3	2.048 (2)	C10—H10	0.9300
O1—C11	1.224 (2)	C12—C13	1.372 (3)
N1—C11	1.367 (3)	C12—C17	1.432 (3)
N1—C12	1.396 (3)	C13—C14	1.405 (4)
N1—H1	0.81 (2)	C13—H13	0.9300
N2—C20	1.316 (2)	C14—C15	1.359 (3)
N2—C17	1.367 (2)	C14—H14	0.9300
C1—C2	1.429 (3)	C15—C16	1.417 (3)
C1—C5	1.435 (3)	C15—H15	0.9300
C1—C11	1.469 (3)	C16—C18	1.402 (3)
C2—C3	1.404 (3)	C16—C17	1.415 (3)
C2—H2	0.9300	C18—C19	1.354 (3)
C3—C4	1.417 (3)	C18—H18	0.9300
C3—H3	0.9300	C19—C20	1.399 (3)
C4—C5	1.412 (3)	C19—H19	0.9300
C4—H4	0.9300	C20—H20	0.9300
C5—H5	0.9300		
			
C10—Fe1—C5	108.35 (10)	C5—C4—H4	125.8
C10—Fe1—C1	120.82 (10)	C3—C4—H4	125.8
C5—Fe1—C1	41.47 (8)	Fe1—C4—H4	126.3
C10—Fe1—C6	40.16 (12)	C4—C5—C1	108.12 (18)
C5—Fe1—C6	126.24 (11)	C4—C5—Fe1	70.04 (12)
C1—Fe1—C6	107.81 (10)	C1—C5—Fe1	69.30 (10)
C10—Fe1—C7	67.67 (11)	C4—C5—H5	125.9
C5—Fe1—C7	164.01 (13)	C1—C5—H5	125.9
C1—Fe1—C7	125.97 (11)	Fe1—C5—H5	126.3
C6—Fe1—C7	40.79 (11)	C10—C6—C7	107.2 (3)
C10—Fe1—C9	39.50 (13)	C10—C6—Fe1	69.85 (15)
C5—Fe1—C9	120.23 (10)	C7—C6—Fe1	69.62 (15)
C1—Fe1—C9	155.04 (10)	C10—C6—H6	126.4
C6—Fe1—C9	67.18 (12)	C7—C6—H6	126.4
C7—Fe1—C9	67.32 (11)	Fe1—C6—H6	125.7
C10—Fe1—C4	126.11 (12)	C8—C7—C6	107.4 (2)
C5—Fe1—C4	40.68 (9)	C8—C7—Fe1	70.25 (14)
C1—Fe1—C4	69.13 (10)	C6—C7—Fe1	69.59 (14)
C6—Fe1—C4	163.18 (11)	C8—C7—H7	126.3
C7—Fe1—C4	154.22 (12)	C6—C7—H7	126.3
C9—Fe1—C4	108.19 (11)	Fe1—C7—H7	125.4
C10—Fe1—C2	156.06 (12)	C9—C8—C7	107.7 (3)
C5—Fe1—C2	68.87 (8)	C9—C8—Fe1	69.94 (14)
C1—Fe1—C2	41.18 (8)	C7—C8—Fe1	69.38 (14)
C6—Fe1—C2	121.07 (11)	C9—C8—H8	126.1
C7—Fe1—C2	108.08 (10)	C7—C8—H8	126.1
C9—Fe1—C2	162.73 (11)	Fe1—C8—H8	126.1
C4—Fe1—C2	68.23 (10)	C10—C9—C8	108.9 (3)
C10—Fe1—C8	66.96 (11)	C10—C9—Fe1	69.91 (14)
C5—Fe1—C8	153.99 (11)	C8—C9—Fe1	70.33 (14)
C1—Fe1—C8	163.43 (10)	C10—C9—H9	125.5
C6—Fe1—C8	67.85 (12)	C8—C9—H9	125.5
C7—Fe1—C8	40.38 (12)	Fe1—C9—H9	125.8
C9—Fe1—C8	39.72 (11)	C9—C10—C6	108.8 (2)
C4—Fe1—C8	119.76 (12)	C9—C10—Fe1	70.60 (15)
C2—Fe1—C8	126.08 (10)	C6—C10—Fe1	70.00 (14)
C10—Fe1—C3	162.78 (13)	C9—C10—H10	125.6
C5—Fe1—C3	68.49 (9)	C6—C10—H10	125.6
C1—Fe1—C3	68.83 (9)	Fe1—C10—H10	125.4
C6—Fe1—C3	155.20 (12)	O1—C11—N1	122.5 (2)
C7—Fe1—C3	120.02 (11)	O1—C11—C1	122.14 (19)
C9—Fe1—C3	126.23 (12)	N1—C11—C1	115.31 (17)
C4—Fe1—C3	40.59 (9)	C13—C12—N1	125.55 (19)
C2—Fe1—C3	40.22 (9)	C13—C12—C17	119.11 (19)
C8—Fe1—C3	107.98 (10)	N1—C12—C17	115.32 (16)
C11—N1—C12	128.71 (18)	C12—C13—C14	120.5 (2)
C11—N1—H1	119.1 (16)	C12—C13—H13	119.7
C12—N1—H1	111.5 (16)	C14—C13—H13	119.7
C20—N2—C17	116.78 (17)	C15—C14—C13	121.7 (2)
C2—C1—C5	106.7 (2)	C15—C14—H14	119.2
C2—C1—C11	123.43 (18)	C13—C14—H14	119.2
C5—C1—C11	129.84 (17)	C14—C15—C16	119.8 (2)
C2—C1—Fe1	69.76 (11)	C14—C15—H15	120.1
C5—C1—Fe1	69.23 (11)	C16—C15—H15	120.1
C11—C1—Fe1	123.63 (14)	C18—C16—C17	117.39 (18)
C3—C2—C1	108.78 (19)	C18—C16—C15	123.4 (2)
C3—C2—Fe1	70.34 (12)	C17—C16—C15	119.2 (2)
C1—C2—Fe1	69.06 (11)	N2—C17—C16	122.62 (17)
C3—C2—H2	125.6	N2—C17—C12	117.68 (17)
C1—C2—H2	125.6	C16—C17—C12	119.70 (17)
Fe1—C2—H2	126.6	C19—C18—C16	119.8 (2)
C2—C3—C4	108.1 (2)	C19—C18—H18	120.1
C2—C3—Fe1	69.44 (12)	C16—C18—H18	120.1
C4—C3—Fe1	69.24 (12)	C18—C19—C20	118.7 (2)
C2—C3—H3	125.9	C18—C19—H19	120.7
C4—C3—H3	125.9	C20—C19—H19	120.7
Fe1—C3—H3	127.0	N2—C20—C19	124.65 (19)
C5—C4—C3	108.3 (2)	N2—C20—H20	117.7
C5—C4—Fe1	69.29 (12)	C19—C20—H20	117.7
C3—C4—Fe1	70.16 (12)		
			
C10—Fe1—C1—C2	159.14 (15)	C5—Fe1—C6—C7	−167.14 (16)
C5—Fe1—C1—C2	−117.82 (19)	C1—Fe1—C6—C7	−124.89 (16)
C6—Fe1—C1—C2	117.14 (16)	C9—Fe1—C6—C7	81.22 (18)
C7—Fe1—C1—C2	75.68 (18)	C4—Fe1—C6—C7	158.4 (3)
C9—Fe1—C1—C2	−168.8 (2)	C2—Fe1—C6—C7	−81.73 (18)
C4—Fe1—C1—C2	−80.40 (15)	C8—Fe1—C6—C7	38.08 (16)
C8—Fe1—C1—C2	45.1 (4)	C3—Fe1—C6—C7	−47.0 (3)
C3—Fe1—C1—C2	−36.77 (13)	C10—C6—C7—C8	−0.3 (3)
C10—Fe1—C1—C5	−83.03 (17)	Fe1—C6—C7—C8	−60.39 (16)
C6—Fe1—C1—C5	−125.04 (14)	C10—C6—C7—Fe1	60.05 (17)
C7—Fe1—C1—C5	−166.50 (15)	C10—Fe1—C7—C8	80.21 (18)
C9—Fe1—C1—C5	−51.0 (3)	C5—Fe1—C7—C8	158.8 (3)
C4—Fe1—C1—C5	37.42 (12)	C1—Fe1—C7—C8	−167.07 (14)
C2—Fe1—C1—C5	117.82 (19)	C6—Fe1—C7—C8	118.2 (2)
C8—Fe1—C1—C5	163.0 (3)	C9—Fe1—C7—C8	37.31 (16)
C3—Fe1—C1—C5	81.05 (14)	C4—Fe1—C7—C8	−47.7 (3)
C10—Fe1—C1—C11	41.8 (2)	C2—Fe1—C7—C8	−124.92 (16)
C5—Fe1—C1—C11	124.8 (2)	C3—Fe1—C7—C8	−82.58 (17)
C6—Fe1—C1—C11	−0.25 (19)	C10—Fe1—C7—C6	−37.94 (17)
C7—Fe1—C1—C11	−41.7 (2)	C5—Fe1—C7—C6	40.7 (4)
C9—Fe1—C1—C11	73.8 (3)	C1—Fe1—C7—C6	74.78 (18)
C4—Fe1—C1—C11	162.22 (18)	C9—Fe1—C7—C6	−80.84 (18)
C2—Fe1—C1—C11	−117.4 (2)	C4—Fe1—C7—C6	−165.9 (2)
C8—Fe1—C1—C11	−72.3 (4)	C2—Fe1—C7—C6	116.93 (17)
C3—Fe1—C1—C11	−154.15 (18)	C8—Fe1—C7—C6	−118.2 (2)
C5—C1—C2—C3	−0.4 (2)	C3—Fe1—C7—C6	159.27 (16)
C11—C1—C2—C3	176.95 (18)	C6—C7—C8—C9	0.3 (3)
Fe1—C1—C2—C3	59.31 (14)	Fe1—C7—C8—C9	−59.66 (17)
C5—C1—C2—Fe1	−59.67 (13)	C6—C7—C8—Fe1	59.97 (16)
C11—C1—C2—Fe1	117.64 (18)	C10—Fe1—C8—C9	36.84 (19)
C10—Fe1—C2—C3	−169.1 (2)	C5—Fe1—C8—C9	−47.9 (3)
C5—Fe1—C2—C3	−81.27 (14)	C1—Fe1—C8—C9	158.4 (3)
C1—Fe1—C2—C3	−120.17 (18)	C6—Fe1—C8—C9	80.49 (19)
C6—Fe1—C2—C3	158.25 (14)	C7—Fe1—C8—C9	118.9 (2)
C7—Fe1—C2—C3	115.40 (16)	C4—Fe1—C8—C9	−82.8 (2)
C9—Fe1—C2—C3	43.8 (4)	C2—Fe1—C8—C9	−166.38 (17)
C4—Fe1—C2—C3	−37.43 (13)	C3—Fe1—C8—C9	−125.57 (18)
C8—Fe1—C2—C3	74.31 (18)	C10—Fe1—C8—C7	−82.11 (19)
C10—Fe1—C2—C1	−48.9 (3)	C5—Fe1—C8—C7	−166.9 (2)
C5—Fe1—C2—C1	38.90 (13)	C1—Fe1—C8—C7	39.4 (4)
C6—Fe1—C2—C1	−81.57 (16)	C6—Fe1—C8—C7	−38.46 (16)
C7—Fe1—C2—C1	−124.43 (16)	C9—Fe1—C8—C7	−118.9 (2)
C9—Fe1—C2—C1	164.0 (3)	C4—Fe1—C8—C7	158.25 (16)
C4—Fe1—C2—C1	82.75 (14)	C2—Fe1—C8—C7	74.68 (19)
C8—Fe1—C2—C1	−165.52 (15)	C3—Fe1—C8—C7	115.49 (17)
C3—Fe1—C2—C1	120.17 (18)	C7—C8—C9—C10	−0.2 (3)
C1—C2—C3—C4	0.1 (2)	Fe1—C8—C9—C10	−59.46 (18)
Fe1—C2—C3—C4	58.59 (15)	C7—C8—C9—Fe1	59.30 (16)
C1—C2—C3—Fe1	−58.52 (14)	C5—Fe1—C9—C10	−82.30 (19)
C10—Fe1—C3—C2	164.9 (3)	C1—Fe1—C9—C10	−45.7 (3)
C5—Fe1—C3—C2	82.30 (13)	C6—Fe1—C9—C10	37.51 (17)
C1—Fe1—C3—C2	37.62 (12)	C7—Fe1—C9—C10	81.92 (19)
C6—Fe1—C3—C2	−49.2 (3)	C4—Fe1—C9—C10	−125.21 (17)
C7—Fe1—C3—C2	−82.63 (16)	C2—Fe1—C9—C10	159.7 (3)
C9—Fe1—C3—C2	−165.24 (14)	C8—Fe1—C9—C10	119.8 (3)
C4—Fe1—C3—C2	119.84 (19)	C3—Fe1—C9—C10	−166.61 (16)
C8—Fe1—C3—C2	−125.11 (15)	C10—Fe1—C9—C8	−119.8 (3)
C10—Fe1—C3—C4	45.1 (4)	C5—Fe1—C9—C8	157.87 (17)
C5—Fe1—C3—C4	−37.54 (14)	C1—Fe1—C9—C8	−165.6 (2)
C1—Fe1—C3—C4	−82.22 (15)	C6—Fe1—C9—C8	−82.32 (19)
C6—Fe1—C3—C4	−169.0 (2)	C7—Fe1—C9—C8	−37.91 (18)
C7—Fe1—C3—C4	157.53 (16)	C4—Fe1—C9—C8	114.96 (18)
C9—Fe1—C3—C4	74.93 (18)	C2—Fe1—C9—C8	39.9 (4)
C2—Fe1—C3—C4	−119.84 (19)	C3—Fe1—C9—C8	73.6 (2)
C8—Fe1—C3—C4	115.05 (16)	C8—C9—C10—C6	−0.1 (3)
C2—C3—C4—C5	0.3 (2)	Fe1—C9—C10—C6	−59.78 (17)
Fe1—C3—C4—C5	58.97 (15)	C8—C9—C10—Fe1	59.72 (18)
C2—C3—C4—Fe1	−58.72 (15)	C7—C6—C10—C9	0.3 (3)
C10—Fe1—C4—C5	75.47 (18)	Fe1—C6—C10—C9	60.15 (18)
C1—Fe1—C4—C5	−38.14 (12)	C7—C6—C10—Fe1	−59.90 (16)
C6—Fe1—C4—C5	44.4 (4)	C5—Fe1—C10—C9	115.56 (16)
C7—Fe1—C4—C5	−169.1 (2)	C1—Fe1—C10—C9	159.40 (15)
C9—Fe1—C4—C5	115.51 (15)	C6—Fe1—C10—C9	−119.5 (2)
C2—Fe1—C4—C5	−82.48 (14)	C7—Fe1—C10—C9	−80.97 (18)
C8—Fe1—C4—C5	157.37 (14)	C4—Fe1—C10—C9	73.90 (19)
C3—Fe1—C4—C5	−119.6 (2)	C2—Fe1—C10—C9	−165.3 (2)
C10—Fe1—C4—C3	−164.95 (16)	C8—Fe1—C10—C9	−37.05 (16)
C5—Fe1—C4—C3	119.6 (2)	C3—Fe1—C10—C9	39.1 (4)
C1—Fe1—C4—C3	81.44 (15)	C5—Fe1—C10—C6	−124.94 (17)
C6—Fe1—C4—C3	164.0 (3)	C1—Fe1—C10—C6	−81.10 (18)
C7—Fe1—C4—C3	−49.6 (3)	C7—Fe1—C10—C6	38.53 (17)
C9—Fe1—C4—C3	−124.92 (16)	C9—Fe1—C10—C6	119.5 (2)
C2—Fe1—C4—C3	37.09 (13)	C4—Fe1—C10—C6	−166.60 (16)
C8—Fe1—C4—C3	−83.05 (17)	C2—Fe1—C10—C6	−45.8 (3)
C3—C4—C5—C1	−0.5 (2)	C8—Fe1—C10—C6	82.45 (19)
Fe1—C4—C5—C1	59.03 (14)	C3—Fe1—C10—C6	158.6 (3)
C3—C4—C5—Fe1	−59.52 (15)	C12—N1—C11—O1	5.3 (3)
C2—C1—C5—C4	0.5 (2)	C12—N1—C11—C1	−174.16 (18)
C11—C1—C5—C4	−176.56 (19)	C2—C1—C11—O1	6.2 (3)
Fe1—C1—C5—C4	−59.49 (15)	C5—C1—C11—O1	−177.1 (2)
C2—C1—C5—Fe1	60.01 (13)	Fe1—C1—C11—O1	92.8 (2)
C11—C1—C5—Fe1	−117.1 (2)	C2—C1—C11—N1	−174.32 (18)
C10—Fe1—C5—C4	−124.51 (17)	C5—C1—C11—N1	2.3 (3)
C1—Fe1—C5—C4	119.40 (18)	Fe1—C1—C11—N1	−87.69 (19)
C6—Fe1—C5—C4	−165.47 (16)	C11—N1—C12—C13	−8.9 (3)
C7—Fe1—C5—C4	162.7 (3)	C11—N1—C12—C17	169.84 (19)
C9—Fe1—C5—C4	−82.90 (17)	N1—C12—C13—C14	179.05 (19)
C2—Fe1—C5—C4	80.77 (15)	C17—C12—C13—C14	0.4 (3)
C8—Fe1—C5—C4	−49.6 (3)	C12—C13—C14—C15	−1.0 (3)
C3—Fe1—C5—C4	37.46 (14)	C13—C14—C15—C16	0.6 (3)
C10—Fe1—C5—C1	116.08 (15)	C14—C15—C16—C18	−178.2 (2)
C6—Fe1—C5—C1	75.13 (16)	C14—C15—C16—C17	0.5 (3)
C7—Fe1—C5—C1	43.3 (4)	C20—N2—C17—C16	1.9 (3)
C9—Fe1—C5—C1	157.69 (15)	C20—N2—C17—C12	−177.45 (17)
C4—Fe1—C5—C1	−119.40 (18)	C18—C16—C17—N2	−1.8 (3)
C2—Fe1—C5—C1	−38.63 (13)	C15—C16—C17—N2	179.49 (18)
C8—Fe1—C5—C1	−169.0 (2)	C18—C16—C17—C12	177.61 (17)
C3—Fe1—C5—C1	−81.94 (14)	C15—C16—C17—C12	−1.1 (3)
C5—Fe1—C6—C10	74.7 (2)	C13—C12—C17—N2	−179.88 (17)
C1—Fe1—C6—C10	116.98 (17)	N1—C12—C17—N2	1.3 (2)
C7—Fe1—C6—C10	−118.1 (2)	C13—C12—C17—C16	0.7 (3)
C9—Fe1—C6—C10	−36.91 (16)	N1—C12—C17—C16	−178.11 (16)
C4—Fe1—C6—C10	40.3 (4)	C17—C16—C18—C19	−0.1 (3)
C2—Fe1—C6—C10	160.14 (15)	C15—C16—C18—C19	178.6 (2)
C8—Fe1—C6—C10	−80.05 (18)	C16—C18—C19—C20	1.7 (3)
C3—Fe1—C6—C10	−165.1 (2)	C17—N2—C20—C19	−0.2 (3)
C10—Fe1—C6—C7	118.1 (2)	C18—C19—C20—N2	−1.6 (3)
